# Next-Generation Beneficial Microbes: The Case of *Akkermansia muciniphila*

**DOI:** 10.3389/fmicb.2017.01765

**Published:** 2017-09-22

**Authors:** Patrice D. Cani, Willem M. de Vos

**Affiliations:** ^1^Walloon Excellence in Life Sciences and Biotechnology (WELBIO), Metabolism and Nutrition Research Group, Louvain Drug Research Institute, Université catholique de Louvain Brussels, Belgium; ^2^Laboratory of Microbiology, Wageningen University Wageningen, Netherlands; ^3^Immunobiology Research Program, Research Programs Unit, Department of Bacteriology and Immunology, University of Helsinki Helsinki, Finland

**Keywords:** *Akkermansia muciniphila*, obesity, diabetes mellitus, type 2, probiotics and prebiotics, gut barrier function

## Abstract

Metabolic disorders associated with obesity and cardiometabolic disorders are worldwide epidemic. Among the different environmental factors, the gut microbiota is now considered as a key player interfering with energy metabolism and host susceptibility to several non-communicable diseases. Among the next-generation beneficial microbes that have been identified, *Akkermansia muciniphila* is a promising candidate. Indeed, *A. muciniphila* is inversely associated with obesity, diabetes, cardiometabolic diseases and low-grade inflammation. Besides the numerous correlations observed, a large body of evidence has demonstrated the causal beneficial impact of this bacterium in a variety of preclinical models. Translating these exciting observations to human would be the next logic step and it now appears that several obstacles that would prevent the use of *A. muciniphila* administration in humans have been overcome. Moreover, several lines of evidence indicate that pasteurization of *A. muciniphila* not only increases its stability but more importantly increases its efficacy. This strongly positions *A. muciniphila* in the forefront of next-generation candidates for developing novel food or pharma supplements with beneficial effects. Finally, a specific protein present on the outer membrane of *A. muciniphila*, termed Amuc_1100, could be strong candidate for future drug development. In conclusion, as plants and its related knowledge, known as pharmacognosy, have been the source for designing drugs over the last century, we propose that microbes and microbiomegnosy, or knowledge of our gut microbiome, can become a novel source of future therapies.

## Introduction

Overweight and obesity have reached epidemic proportions with more than 600 million of adults and 100 million children of the world’s population suffering from obesity (GBD 2015 Obesity Collaborators et al., 2017). Obesity predisposes to the development of type 2 diabetes and cardiovascular diseases. These two pathologies are part of the metabolic syndrome that is also becoming major problem in public health (Abdelaal et al., 2017; Ajala et al., 2017). Gut microbes play an important role in the regulation of host metabolism and low-grade inflammation (Hartstra et al., 2015; Marchesi et al., 2016; Cani, 2017). The perturbation of the composition and the activity of the gut microbiota, also known as dysbiosis, is thought to be involved in the emergence of the metabolic syndrome (Wen and Duffy, 2017). Nowadays, numerous studies have demonstrated that our dietary habits strongly influence the composition and function of the gut microbiota and eventually may contribute to the onset or the protection against metabolic disorders (David et al., 2014; Korpela et al., 2014; Salonen et al., 2014; Carmody et al., 2015; Zeevi et al., 2015; Cani and Everard, 2016; Thaiss et al., 2016).

Well documented among the potential ways to affect the gut microbiota, is the consumption of selected microbes that are marketed as probiotics defined as “live microorganisms that when administered in adequate amounts confer a health benefit on the host” (Hill et al., 2014). It is worth noting that the current majority of probiotics sold on the market include mainly microorganisms from the genera *Lactobacillus* and *Bifidobacterium* (Douillard and de Vos, 2014). However, other ways such as the consumption of prebiotics have gained considerable attention over the last 20 years (Roberfroid et al., 2010). The prebiotic concept, discovered in Gibson and Roberfroid (1995), has led to a great number of dietary supplements that is an important growth market. The definition of prebiotic is now widely used and has been recently revised as “a substrate that is selectively utilized by host microorganisms conferring a health benefit” (Gibson et al., 2017). Thus, nutritional components that escape the digestion in the upper alimentary tract may have an impact on the gut microbiota by modulating some members of the gut microbiota its composition and its activity. However, the concept of prebiotic has not yet revealed all its secrets. In spite of numerous discoveries of molecular mechanisms explaining how prebiotics and the gut microbiota interact with the host, it remains difficult to identify the bacterial candidate(s) involved in the beneficial effects observed on the energy, glucose, lipid metabolism and immunity.

## From Prebiotic to Next-Generation Beneficial Microbe: Focus on the Identification of *Akkermansia muciniphila*

*Akkermansia muciniphila* is one of the most abundant single species in the human intestinal microbiota (0.5–5% of the total bacteria) and has been isolated and characterized as a mucin-utilizing specialist in 2004 by Muriel Derrien in her Ph.D. research at Wageningen University (Derrien et al., 2004; Collado et al., 2007). This discovery was initiated by the notion that the human body produces its own “prebiotics” or microbial substrates, namely mucus, an abundant glycoprotein that is specifically produced and degraded in the colon (Ouwehand et al., 2005). While germ-free mouse experiments showed that *A. muciniphila* showed immune and metabolic signaling, specifically in the colon, the exact functions of this unusual microbe remained enigmatic (Derrien et al., 2008, 2011).

**FIGURE 1:**
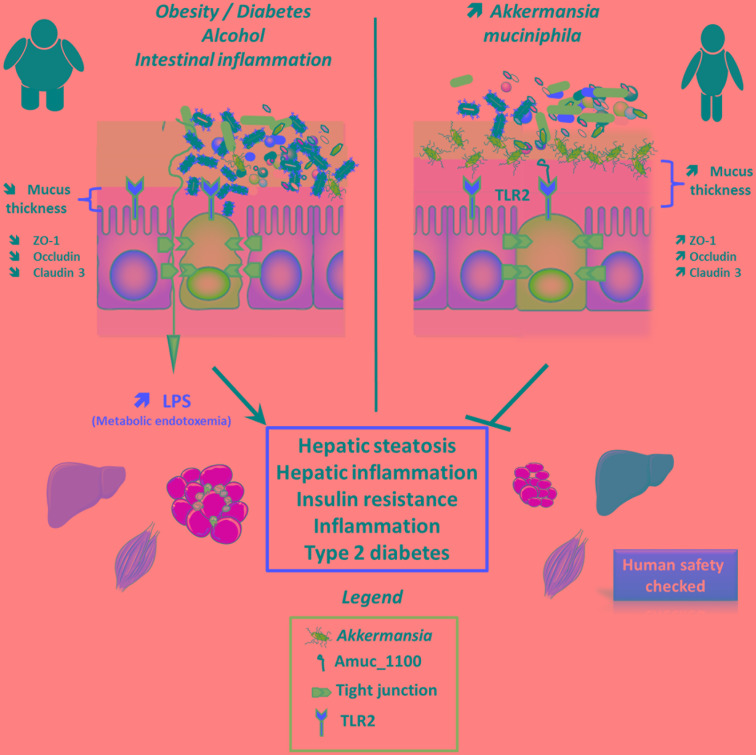
Effects of *A. muciniphila* and derived products on host metabolism. *A. muciniphila* has been found to be lower in several conditions such as during obesity, diabetes, intestinal inflammation, liver diseases, or chronic alcohol consumption. This is associated with an altered gut barrier function leading to an increased plasma LPS levels and eventually triggering low grade inflammation and metabolic disorders. *A. muciniphila* alive or pasteurized as well as Amuc_1100 has been shown to restore gut barrier function likely by acting on TLR2 and restoring appropriate tight junction expression. All these results are associated with an increased mucus later thickness and an improvement of metabolic disorders. It is worth noting that an exploratory human investigation has shown that *A. muciniphila* is apparently safe.

Further indications for the function of *A. muciniphila* were subsequently determined in other prebiotic studies using inulin-type fructans that were initially characterized as bifidogenic compounds able to increase the abundance of *Bifidobacterium* spp. (Gibson and Roberfroid, 1995). Thanks to the development of novel culture-independent techniques, we decided to revise in depth the impact of such kind of prebiotics on the overall microbial community in mice. Therefore, in search of potential novel bacterial candidates, we combined different techniques (phylogenetic microarray, high-throughput sequencing, gradient denaturation gel and qPCR), which allowed us to analyze and to compare all the bacteria that were present in the intestinal microbiota. The first surprise was to discover that more than 100 different taxa were affected by prebiotics (Everard et al., 2011; Everard et al., 2014). Among these bacteria, we found that the relative abundance of *A. muciniphila* increased more than 100-fold following the ingestion of prebiotics thereby reaching the abundance of up to 4.5% under high-fat diet (Everard et al., 2014), whereas this effect was lower under normal chow diet (0.09–2.5%) depending on the model (Everard et al., 2011, 2014). It is worth noting that these findings are confirmed in different set of experiments (Everard et al., 2013; Liu et al., 2016; Reid et al., 2016; Catry et al., 2017; Zhu et al., 2017). Interestingly, we and others discovered that *A. muciniphila* was less abundant in the intestinal microbiota of both genetic and diet-induced obese and diabetic mice (Everard et al., 2011, 2013, 2014; Schneeberger et al., 2015; Leal-Diaz et al., 2016; Ojo et al., 2016; Song et al., 2016; Singh et al., 2017), however, few studies reported in mice an increased abundance of *A. muciniphila* upon the ingestion of a high-fat high sucrose diet (Anhe et al., 2015; Carmody et al., 2015). It has also been largely demonstrated that inulin-type fructans feeding improves metabolic disorders associated with obesity, including a decreased fat mass, insulin resistance, lower liver steatosis and a reinforcement of the gut barrier (**Figure 1**) (Cani et al., 2004, 2006, 2009; Maurer et al., 2010; Everard et al., 2011; Pachikian et al., 2012; Greer et al., 2016). Importantly, in humans the abundance of *A. muciniphila* was decreased in several pathological situations such as obesity, type 2 diabetes, inflammatory bowel diseases, hypertension and liver diseases (Png et al., 2010; Belzer and de Vos, 2012; Zhang et al., 2013; Dao et al., 2015; Yassour et al., 2016; Grander et al., 2017; Li et al., 2017). Conversely, antidiabetic treatments, such as metformin administration and bariatric surgery were both found to be associated with a marked increase in the abundance of *A. muciniphila* (**Figure 1**) (Shin et al., 2014; Forslund et al., 2015; de la Cuesta-Zuluaga et al., 2017). Therefore, a large body of evidence suggested that *A. muciniphila* may contribute to protect from specific metabolic disorders and cardiometabolic risk factors associated with a low-grade inflammatory tone.

## Administration of *Akkermansia muciniphila*: Multiple Effects on the gut and Beyond

Inspired by the numerous indications that the relative levels of *A. muciniphila* decreased during obesity and metabolic disorders in mouse and man, we decided to study the causal link between *A. muciniphila* and improvements in metabolism. This was done by investigating the impact of a daily oral supplementation with live *A. muciniphila* on the onset of obesity, diabetes and gut barrier dysfunction in mice. We found that the administration of live *A. muciniphila* at the dose of 2.10^8^ bacterial cells per day was partly protecting against diet-induced obesity in mice (Everard et al., 2013). Indeed, mice showed a 50% lower body weight gain when treated with live *A. muciniphila* without altering neither their dietary food intake nor the elimination of dietary fats in fecal matter. This protection was mirrored by two times less visceral and subcutaneous fat mass (**Figure 1**), but also by increased markers of fatty acid oxidation in the adipose tissue (Everard et al., 2013). In addition, animals receiving live *A. muciniphila* did no longer exhibited insulin resistance, nor infiltration of inflammatory cells (CD11c) in the adipose tissue, which is a key characteristic of obesity and associated low-grade inflammation (Everard et al., 2013). Interestingly, most of all the metabolic improvements observed following treatment with live *A. muciniphila* were in the range as those observed following oligofructose or inulin treatment (Cani et al., 2009; Dewulf et al., 2011; Everard et al., 2011, 2014), although live *A. muciniphila* was not affecting food intake behavior as do prebiotics like inulin and oligofructose.

Knowing that these metabolic features can be caused by an increased plasma LPS level (i.e., metabolic endotoxemia) or bacterial translocation (Cani et al., 2007; Amar et al., 2011), we next investigated the gut barrier function by measuring several markers. We observed that live *A. muciniphila* prevented the development of metabolic endotoxemia, an effect associated with the restoration of a normal mucus layer thickness (**Figure 1**) (Everard et al., 2013). We also found that administration of live *A. muciniphila* restored the endogenous production of antimicrobial peptides. We then discovered that live *A. muciniphila* increased the endogenous production of specific bioactive lipids that belongs to the endocannabinoid family and are known to have anti-inflammatory activities and regulating the endogenous production of gut peptides involved in glucose regulation and gut barrier, respectively, glucagon-like peptide-1 and 2 (GLP-1 and GLP-2) (Cani et al., 2016). It is worth noting that all these findings have subsequently been confirmed by different groups and extended to other specific disorders such as atherosclerosis, hepatic inflammation and hypercholesterolemia (Shin et al., 2014; Li et al., 2016; Shen et al., 2016; Grander et al., 2017; Plovier et al., 2017).

Collectively all these data reinforce the assumption that live *A. muciniphila* can be considered as a next-generation beneficial microbe with the exceptional particularity that this bacterium can act on numerous facets of the metabolic syndrome and cardiometabolic disorders. Still, these discoveries have raised different fundamental questions that will still have to be studied in humans with the aim to generate new therapeutic tools.

## Crossing the Barrier of Species: from Mice to Man

*Akkermansia muciniphila* requires specific culture conditions and complex animal-based medium (i.e., mucin from animal source) and although it may respire under microaerophilic conditions, the cells are relatively sensitive to oxygen (Ouwerkerk et al., 2016). These properties complicate the administration of *A. muciniphila* to human as to evaluate its potential, hence limiting its therapeutic perspectives. In order to solve this problem, a synthetic medium was developed in order to allow the culture of *A. muciniphila* with a high yield and devoid of compounds incompatible with administration in humans (Plovier et al., 2017; Van der Ark et al., unpublished data). Besides the successful development of this synthetic medium, the previous assessment of the efficacy of *A. muciniphila* were performed with cells grown on a mucin-based medium. Therefore, the bacteria cultured on the different media were tested and compared. Interestingly, *A. muciniphila* retains its effectiveness independently of the medium used, and as previously observed, mice treated with the bacterium gained less weight, exhibited an improved glucose tolerance, and insulin resistance under hyperlipidic diet (**Figure 1**) (Plovier et al., 2017).

## Serendipity: the Unexpected Advantage of Pasteurization

In 2013, it was showed that the protective effects of *A. muciniphila* disappeared when the bacterium was destroyed by using autoclaving, a heat treatment that destroyes all the constituents of bacteria and spores (Everard et al., 2013). As *A. muciniphila* is a Gram-negative bacterium and hence no spore-former, we were interested what the effects would be of pasteurization, a milder heat inactivation method than autoclaving. Therefore, we tested the impact of administrating pasteurized *A. muciniphila* (30 min at 70°C) cells on diet-induced metabolic disorders in mice. Unexpectedly, this method of inactivation did not abolish the effect of *A. muciniphila* but even exacerbated its beneficial impact. Specifically, mice receiving the pasteurized bacterium and the high-fat diet had a similar body weight gain and fat mass than those observed in mice fed a control diet. Again, these effects were independent of the food intake but pasteurized *A. muciniphila* increased the loss of energy in the feces of the treated mice, indicating a decrease in energy absorption that could contribute to explain the lower weight gain. Pasteurized *A. muciniphila* also strongly improved glucose tolerance, hepatic insulin sensitivity, and completely blocked the diet-induced metabolic endotoxemia. Although, the mechanisms of action of the bacteria are not yet fully elucidated, it is known that *A. muciniphila* express numerous highly abundant protein on its outer membrane (Ottman et al., 2017). Among these proteins, Amuc_1100, implicated in the formation of pili by *A. muciniphila*, was one of the most abundant (Plovier et al., 2017).

## *Akkermansia muciniphila*: A Gate Keeper that Dialogs with the Innate Immune System

We previously found that *A. muciniphila* was able to restore the expression of specific antimicrobial peptides (Everard et al., 2013). However, Nucleotide oligomerization domain (NOD)-like receptors (NLRs) and Toll-Like Receptors (TLRs) are a specialized group of membrane and intracellular proteins that play a critical role in the regulation of immunity and are directly involved in the recognition of bacterial constituents by the immune system. Therefore, we evaluated the potential of *A. muciniphila* to activate different NOD and TLRs. Strikingly, we found that the bacteria specifically interact with TLR2. TLR2 has been shown to modulate intestinal homeostasis and host metabolism (Caricilli et al., 2011; Brun et al., 2013), thereby participating in the interactions between microbes and host. In addition, to better characterize the interaction between *A. muciniphila* and this receptor, we took advantage of genomic and proteomic analyzes of the external membrane of the bacterium, which may be exposed to host receptors (Ottman et al., 2016). Among these proteins, Amuc_1100 was one of the most abundant. This protein is implicated in the formation of pili by *A. muciniphila* and thus could participate in the interaction between the bacterium and TLR2. This hypothesis was further confirmed by showing that a version of the genetically engineered protein (called Amuc_1100^∗^) was effectively activating TLR2 and with the same magnitude as *A. muciniphila*. In addition, Amuc_1100^∗^ remained stable at the temperature used during pasteurization, and could therefore contribute to the effects of the pasteurized bacterium. Amuc_1100^∗^ was also able to replicate almost all the effects of *A. muciniphila* alive or pasteurized in high-fat diet fed mice. *A. muciniphila*, whether live or pasteurized, and Amuc_1100^∗^ also decreased high cholesterol levels induced by the high-fat diet. Conversely, the pasteurized bacterium specifically also reduced the triglyceridemia of the treated mice, reinforcing the idea that the pasteurization of *A. muciniphila* reinforces its protective effects. A potential mechanism explaining this could be the exposure of active molecules by the heat treatments, including Amuc_1100, or the inactivation of inhibitory compounds, or combinations thereof.

## First Assessment of *Akkermansia muciniphila* in Humans with Metabolic Syndrome

As discussed earlier, *A. muciniphila* has various advantages as compared to other beneficial microbes and specific probiotics, at least in the context of the metabolic syndrome. *A. muciniphila* is present in the human milk, is highly abundant in lean and non-diabetic subjects, and is even highly increased upon metformin treatment of gastric bypass surgery, and this without obvious deleterious impact. This unique character does not preclude the fact the human investigations and safety assessment must be done. Hence, to become a putative future food supplement, the safety must be tested. We evaluated the toxicity and the emergence of possible side effects related to the administration of *A. muciniphila* in humans (20 subjects) as part of an ongoing clinical trial of individuals with metabolic syndrome (Plovier et al., 2017). To this end, we analyzed relevant clinical parameters related to liver, muscles and renal functions as well as markers of immunity and inflammation in individuals who received *A. muciniphila* daily for 2 weeks and then extended to 3 months. Whatever the formulation of *A. muciniphila* (live at 10^9^ and 10^10^ bacteria per day or pasteurized at 10^10^ bacteria per day), no changes were observed for the markers tested after 2 weeks or 3 months of daily administration. In addition, the frequency of side effects reported by patients were similar in the different groups. These first data indicate that *A. muciniphila* (active or pasteurized) is tolerated in individuals with metabolic syndrome and is likely not toxic.

While *A. muciniphila* is one of the handful of core microbes identified in the intestinal microbiota of over 1000 human adults (Shetty et al., 2017), the administration of its cells, either in live or pasteurized form, in a dietary supplement may be subject to regulatory frameworks that aim to safeguard the consumer. The regulatory requirements relating to the use of live *A. muciniphila* have recently been addressed (Gomez-Gallego et al., 2016). This review summarized the recent comprehensive studies related to *A. muciniphila* and its safety properties and provided criteria be addressed when *A. muciniphila* cells are to be considered as a novel food by the European Food Safety Authority in Europe. One aspect that is relevant here and applies to other core intestinal microbes as well, is the fact that most if not all healthy subjects carry these anaerobes. So these have to be consumed at some stage and in this context it is important to note that *A. muciniphila* is present in early life microbiota and has been detected in mothers’ milk (Collado et al., 2007, 2012; Derrien et al., 2008; Jeurink et al., 2013; Ward et al., 2013). Another aspect relates to the antibiotic resistance of *A. muciniphila* that has been studied to some extent in healthy human subjects that carried high levels of *A. muciniphila*-like bacteria and apparently were sensitive to penicillin and tetracycline derivatives but resistant to vancomycin (Dubourg et al., 2013). This was confirmed in *in vitro* studies on the antibiotic resistance profile with the type strain Amuc^T^ (Ouwerkerk Ph.D. Thesis Wageningen University 2016). Moreover, inspection of the genome sequence did not reveal antibiotic resistance genes that are linked to known genetically transferrable elements (Gomez-Gallego et al., 2016).

## Conclusion

Since its discovery in 2004, numerous studies have mostly linked the abundance of *A. muciniphila* with beneficial effects, and this although very few exceptions exist in specific non-physiological models (i.e., gnotobiotic models, specific immune double knock-out models) (Seregin et al., 2017).

Nowadays, *A. muciniphila* is widely considered as a novel potential candidate to improve metabolic disorders associated with obesity, diabetes, liver diseases and cardiometabolic disorders. Indeed, its administration has been shown to profoundly reduce the development of such diseases.

Other important steps toward the development of *A. muciniphila* as a next-generation beneficial microbe have been successfully reached. First, the discovery that *A. muciniphila* remained effective by being grown on a synthetic medium compatible with administration in humans. Second, the discovery that inactivation of the bacteria by pasteurization improved its effects, and thus its stability and potential shelf life. Third, the identification of a key mechanisms of interaction between *A. muciniphila* and its host via the identification of Amuc_1100, and last but not least; fourth, the demonstration that *A. muciniphila* may be safely administered in the human targeted population.

Finally, the pasteurized bacteria and the identification and the isolation of bacterial constituents such as the relatively small 30-kDa Amuc_1100 open the door to putative development of drugs based on *A. muciniphila*-related product that could also target pathologies such as type 1 diabetes, inflammatory bowel diseases or diseases where the intestinal barrier function is compromised.

## Author Contributions

PC and WdV: Conceptualized the review content.

## Conflict of Interest Statement

PC and WdV are inventors on patent applications dealing with the use of *A. muciniphila* and its components in the treatment of obesity and related disorders. The authors declare that the research was conducted in the absence of any commercial or financial relationships that could be construed as a potential conflict of interest.
